# Influencing factors of home hospice care needs of family caregivers of the older adult with chronic diseases at the end of life in China: a cross-sectional study

**DOI:** 10.3389/fpubh.2024.1348285

**Published:** 2024-05-02

**Authors:** Lei Wang, Yaru Li, Rui Zhao, Jiangxu Li, Xiangru Gong, Hongyu Li

**Affiliations:** College of Nursing, Jinzhou Medical University, Jinzhou, China

**Keywords:** hospice care, cross-sectional studies, older adults, chronic diseases, family caregivers, end of life

## Abstract

**Introduction:**

With increased life expectancy in the Chinese population coupled with chronic disease the care needs of people at the end of life are attracting much attention. Home hospice care can help the dying older adult achieve comfort and maintain their dignity at home. However, dying at home means great responsibility and challenge for family caregivers, and there are many unmet needs. The study aimed to investigate the home hospice care needs of family caregivers of older adult people with chronic diseases at the end of life in China, and to analyze the influencing factors of home hospice care needs of caregivers.

**Methods:**

In this cross-sectional study, from May to September 2023, 4 community health service centers were selected by stratified sampling from seven administrative districts in Jinzhou City, Liaoning Province, where home hospice care was piloted. Then 224 family caregivers were selected from the communities of seven community service centers by simple random sampling method. A general information questionnaire and the home hospice care needs questionnaire developed by our research group were used to investigate. Univariate analysis was used to compare the differences in the scores of different characteristics, and the factors with significant differences were selected for multivariate linear regression analysis to determine the final influencing factors.

**Results:**

The total score of hospice care needs of family caregivers was 121.61 ± 15.24, among which the end-of-life knowledge need dimension score was 24.04 ± 2.71, the highest score index was 80.13%, while the symptom control need score was 15.58 ± 3.39, the lowest score index was 62.32%. In addition, Caregivers with caregiving experience, dying older adult with longer disease duration, and dying older adult with higher levels of education were the factors influencing the total need for home hospice care among family caregivers, with a variance explained of 22.7%.

**Discussion:**

The needs of family caregivers of the terminally ill older adult are high, and healthcare professionals should implement services to meet their multidimensional needs and improve the quality of care according to the factors affecting their needs.

## 1 Introduction

Chronic diseases are defined as non-communicable diseases with long course and slow progress, which are characterized by wide prevalence, high cost, high disability and mortality (1). The most common chronic diseases include stroke, ischemic heart disease, cancer and chronic obstructive pulmonary disease (2). Increasing age is the main risk factor for increasing morbidity and mortality of most chronic diseases (3). China already has the largest older adult population in the world, with approximately 264 million people aged 60 years and older, accounting for 18.7% of the total population (4). It is expected that by 2050 China's total older adult population will reach 400 million, with an aging rate of more than 30% (5). With increased life expectancy in the Chinese population coupled with chronic disease the care needs of people at the end of life are attracting much attention.

Most older adult patients who are dying prefer to get hospice care at home, likely due to the influence of traditional cultural notions in China (6). Home hospice provides palliative and supportive care to terminally ill patients and their families living at home by a team of health-care professionals and social volunteers. Home hospice care allows the dying older adult to achieve comfort and dignity at home, reduces hospitalization time and treatment costs, and improves the quality of death (7). For family caregivers, a death at home involves a great deal of responsibility and challenges. The term “family caregiver” describes the family members who live with the patient and spend the most time caring for the patient or who have the primary responsibility for most of the caregiving tasks, including spouse, child, parent, etc. (8, 9). In the final stages of life, caregivers are in charge of providing the older adult with emotional support, symptom management, and dietary support. Compared with the older adult at the end of life, caregivers lack support, have considerable caregiver burden and many unmet needs. Research indicates that caregivers usually provide care services to patients for ~8 h per day, and when caregiver needs or problems are not met or resolved, it can negatively affect the quality of life of both the patient and the caregiver (10). Therefore, it is of great practical significance to study the home hospice needs of caregivers, and the first step in meeting the needs of family caregivers is to assess their needs.

The research shows that there are many and rich contents on the home hospice care needs of family caregivers in Western countries. The needs of family caregivers include two main aspects: one is the need to take care of dying patients (such as symptom management and end-of-life planning including advanced decision-making and financial planning), and the other is their own physiological and psychological needs when they assume the role of caregivers (such as respite care and psychological counseling) (11–13). Furthermore, Nicolas' systematic scoping review reported that caregivers had the highest unmet needs in the domains of Psychological, Patient Care, and Support (14), and other literature reviews have identified the existence of multiple domains of unmet needs for caregivers, including information needs, and psychological needs (15). In contrast, the development of home hospice care in China has been slow, and research on needs is very limited. Distinct cultural and religious convictions significantly influence hospice needs and the process of making end-of-life decisions (15, 16). Thus, it is necessary to further explore the needs of Chinese family caregivers for home hospice care.

Higher need scores were identified in a cross-sectional study for younger, better educated, and higher household income caregivers (17). Badr et al. hypothesized that poorer physical health of caregivers may increase the unmet needs of caregivers (18). According to Jansma, caregivers with caregiving experience had significantly higher needs for symptom control than those without caregiving experience (19). It is clear that family caregivers' needs for home hospice vary depending on a variety of general information and caregiving-related characteristics. In addition, research has also indicated that factors such as gender, medical burden and self-care ability of terminal cancer patients have an impact on the needs of family caregivers (20), but there are fewer studies on the effects of basic characteristics of older adult terminal patients with chronic diseases on the level of caregiver needs.

This study took the family caregivers of the older adult with chronic diseases at the end of life as the object, and explored its influencing factors based on the survey on the current situation of caregivers' needs, in order to serve as a guide for the community medical staff implementing demand-oriented home hospice interventions.

## 2 Method

### 2.1 Design and samples

From May to September 2023, stratified sampling was conducted in seven administrative districts of Jinzhou City, Liaoning Province, where home hospice care pilot units were carried out, and a total of four community health service centers were selected. Then, a simple random sampling method was used to select 235 family caregivers of terminally ill older adult with chronic diseases who met the inclusion and exclusion criteria from the communities of four community service centers. The Jinzhou Medical University College of Nursing Ethics Committee gave its approval to the study (No. JZMULL2023159).

### 2.2 Inclusion criteria

#### 2.2.1 Older adult with chronic diseases at the end of life

1. Age ≥ 60 years.
2. Chronic non-tumor diseases, with 1 or more organs severely impaired in function, no effective means of cure into the terminal stage or patients with malignant tumors into the terminal stage (expected survival ≤ 6 months).
3. Choose to die at home (hospice).
4. Clear consciousness, with normal language communication, and understanding ability.

#### 2.2.2 Family caregiver

1. Immediate family members (including parents, spouses, children, siblings, etc.) or primary caregivers (the duration of care was 1 month and above).
2. Age ≥18 years old, informed about the condition and involved in nursing decision-making.
3. If there are multiple caregivers taking turns to take care of the patient, the primary caregiver was preferred.

### 2.3 Exclusion criteria

Caregivers those with a history of mental illness or communication disorders; those who with employment relationships; or those who with recent negative stress events (e.g., loss of relatives, car accidents); not completing the questionnaires or completing them incompletely.

### 2.4 Instruments

#### 2.4.1 General information questionnaire

Information collected on the dying older adult included demographic characteristics (gender, age, marital status, education level, medical insurance payment method) and their disease status (duration of illness, number of diseases, the type of disease, self-care ability), totaling six items. Family caregivers included demographic information (e.g., gender, age, marital status, education level) and caregiving status (e.g., caregiving burden, relationship with the dying older adult, length of caregiving), totaling 13 items. Demographics of the dying older adult and family caregivers were collected by self-report, and duration of illness and the type of disease were collected from medical record review.

#### 2.4.2 The family caregiver needs assessment questionnaire for home hospice care of the dying older adult

FCNQ was an assessment tool developed by our research group to assess the home hospice care needs of family caregivers of the dying older adult in China. The development phase of the questionnaire consisted of three steps. First, an initial questionnaire containing 48 items was initially developed based on family caregiver interviews and an extensive review of the literature. Second, the initial items developed and compiled by the researcher were reviewed by two rounds of 19 experts (one clinical geriatric medicine, three chronic disease nursing education, four community chronic disease nursing, four community geriatric nursing, six geriatric hospice care, and one community oncology) was revised to 36 items. Finally, 223 family caregivers of terminally ill older adult were selected for questionnaire validation. The validation process consisted of (1) further screening of 34 items using item analysis and (2) psychometric techniques for reliability and validity analysis. The questionnaire has been psychologically validated and found to have good reliability and validity. The Cronbach's alpha coefficient of the total questionnaire was 0.910, the Cronbach's alpha coefficients of the dimensions were 0.888–0.922, and the retest reliability was 0.868. The content validity index of the questionnaire was 0.982, and the content validity indices of the items were 0.83–1.00. For the exploratory factor extraction of the six common factors, the cumulative variance contribution rate was 71.181%. The questionnaire consisted of the following items: symptom control needs (five items), life care needs (seven items), emotional regulation needs (seven items), social support needs (five items), end-of-life knowledge needs (six items) and spiritual care needs (four items), totaling 34 items. Each item was rated on a 5-point Likert scale from “not needed” to “very needed” on a scale of 1–5. The total questionnaire score ranged from 34 to 170, with higher scores indicating a higher level of need. A score of < 92 was a low need, 92–110 was a medium need, and >110 was a high need. Since the number of items in each dimension is different, in order to make them comparable, the score indicator was used for analysis, which was calculated as follows: score indicator = (the actual score of the dimension/the theoretical maximum score of the dimension) × 100% (21).

### 2.5 Data collection

A research team was set up. The postgraduate tutor was responsible for contacting the community health service center where the survey was conducted and coordinating the time of the door-to-door survey with the community committee. An associate professor was responsible for the quality control of the data collection process, and two PhDs guided the questionnaire design and refinement and were responsible for the training of the questionnaire survey. Two postgraduate students were uniformly trained to collect the questionnaires, and the questionnaires were carried out after passing the training. Before the survey, the purpose and significance of the study were explained to the respondents, and an informed consent form was signed. Then we explained the requirements of filling in the questionnaire and asked the respondents to fill in the questionnaire after they fully understood it. For those with low education levels or unable to answer for other reasons, the researcher read out the questions and options in a uniform way and recorded the actual answers of the respondents. The time limit for answering the questionnaire was 20 min or less.

### 2.6 Statistical analyses

Epidata 3.1 software was used to input the original values by two people and SPSS22.0 (IBM Corp, Armonk, NY) software was used for statistical analysis of data. The two-sided test level was α = 0.05, and the difference was statistically significant at *P* < 0.05.

The frequency and component ratios were used to describe the general information about the older adult with end-stage chronic illness and family caregivers, and the total FCNQ need scores and the scores on each dimension were described by the mean and standard deviation. To analyze differences, first, independent samples *t*-tests (variables were divided into two groups) and one-way ANOVA (variables were divided into three or more groups) were used to initially explore the relationship between the scores on the dimensions of need for home hospice care and general information about study participants. Second, taking the total score and the scores of six dimensions of FCNQ as the dependent variables, the statistically significant independent variables in the single factor analysis were selected for multiple linear regression analyses (α _in_ = 0.05, α _out_ = 0.10) to find out the possible influencing factors of the total needs and the needs of the dimensions.

## 3 Results

### 3.1 General information

A total of 235 questionnaires were distributed in this study, and 224 meeting the requirements were recovered, with a response rate of 95.3%. Eleven invalid questionnaires were excluded, among which six refused to answer part of the questions in the questionnaire due to personal reasons, resulting in incomplete questionnaires. Five were excluded because the subjects filled in the information incorrectly.

The general information on the older adult dying of chronic diseases and their family caregivers are shown in Tables 1, 2. More than half of the dying older adult were male (54.0%), most of them were aged 60–69 years (42.0%), married/ cohabitation (78.6%), and had an education level of primary school or below (56.7%). The most common type of disease was cancer (54.70%), with a high prevalence of lung cancer (21.9%). Among non-cancer chronic diseases (49.1%), the chronic prevalence of cardiovascular diseases was higher (20.1%).

**Table 1:** General Information of older adult with end-stage chronic disease (*N* = 224).

**Characteristics**		***N***	**Percentage (%)**
Gender	Male	121	54.0
	Female	103	46.0
Age (year)	60–69	94	42.0
	70–79	86	38.4
	≥80	44	19.6
Marital status	Unmarried	3	1.3
	Married/cohabitation	176	78.6
	Divorce	4	1.8
	Widowed	41	18.3
Education level	Primary school and below	127	56.7
	Middle school education	67	29.9
	High school education or technical secondary school	13	5.8
	Junior college and above	17	7.6
Medical insurance payment method	Insurance for urban workers	119	53.1
	New rural co-operative medical system	85	37.9
	Commercial health insurance	6	2.7
	Self-paying	14	6.3
Duration of illness (year)	< 1	73	32.6
	1–3	82	36.6
	>3	69	30.8
Number of comorbidities (kind)	1	115	51.3
	2	74	33.0
	≥3	35	15.6
The type of disease	Non-cancer chronic diseases	110	49.1
	Cardiovascular diseases	45	20.1
	Chronic respiratory diseases	30	13.4
	Nervous system diseases	15	6.7
	Cerebrovascular diseases	12	5.4
	Other	8	3.5
	Cancer	114	50.9
	Lung cancer	49	21.9
	Liver cancer	25	11.2
	Colorectal cancer	18	8.0
	Leukemia	9	4.0
	Esophageal cancer	8	3.6
	Other	5	2.2
Self-care ability	Completely capable of	58	25.9
	Most able to	88	39.3
	A part of	50	22.3
	Completely unable	28	12.5

**Table 2:** General information of family caregiver (*N* = 224).

**Characteristics**		***N***	**Percentage (%)**
Gender	Male	85	37.9
	Female	139	62.1
Age (year)	18–40	58	25.9
	41–59	97	43.3
	≥60	69	30.8
Marital status	Unmarried	10	4.5
	Married/cohabitation	212	94.6
	Divorce	2	0.9
Education level	Junior high school and below	133	59.4
	High school or junior college	45	20.1
	College or above	46	20.5
Health status	Very good	89	39.7
	Normal	71	31.7
	Not good enough	48	21.4
	Poor	16	7.1
Religious belief	No	207	92.4
	Yes	17	7.6
Caregiving burden	No burden of care	33	14.7
	A little burden of care	109	48.7
	Heavy burden of care	82	36.6
Relationship with the dying older adult	Spouse	77	34.4
	Children	126	56.5
	Grandchildren	7	3.1
	Sibling	9	4.0
	Other	5	2.2
Length of caregiving (month)	1–6	112	50.0
	6–12	52	23.2
	>12	60	26.8
Household incomes per capita (yuan)	< 3,000	94	42.0
	3,000–4,999	87	38.8
	≥5000	43	19.2
Caregiving experience	No	168	75.0
	Yes	56	25.0
Working condition	Working	116	51.8
	Non-working	108	48.2
Burden of payment for treatment	No burden	15	6.7
	A little burden	56	25.0
	Moderate burden	49	21.9
	Heavy burden	104	46.4

Most of the family caregivers were female (62.1%), married/Cohabitation (94.6%), educated in junior high school and below (59.4%), and non-religious (92.4%). The highest percentage of caregiver-older adult relationships were with Children (56.5%), nearly half of the caregivers had a per capita monthly household income of < 3,000 yuan (42.0%) and had a heavy financial burden of treatment (46.4%).

### 3.2 Family caregiver home hospice needs score

Family caregivers' home hospice needs had a total score of 121.61 ± 15.24, and the score indicator was 71.54%. The score of End-of-life Knowledge Needs was 24.04 ± 2.71, and its score indicator was the highest (80.13%). Spiritual Care Needs (14.62 ± 2.20) and Emotional Regulation Needs (25.44 ± 4.94) followed with score indicators of 73.10 and 72.69%, respectively. Symptom Control Needs with a score of 15.58 ± 3.39 had the lowest score indicator (62.32%). More detailed information is shown in Table 3.

**Table 3:** Scores for items on dimensions of need for home hospice care for family caregiver.

**Dimension**	**Range of score**	**Total score ($\bar{\text{x}}$ ±SD)**	**Index of score (%)**	**Order**
Symptom control needs	5–25	15.58 ± 3.39	62.32	6
Life care needs	7–35	24.35 ± 4.33	69.57	5
Emotional regulation needs	7–35	25.44 ± 4.94	72.69	3
Social support needs	5–25	17.58 ± 3.07	70.32	4
End-of-life knowledge needs	6–30	24.04 ± 2.71	80.13	1
Spiritual care needs	4–20	14.62 ± 2.20	73.10	2
Total score	34–170	121.61 ± 15.24	71.54	

Table 4 lists the five highest scoring items for each dimension. It involved multiple dimensions. The top two need items were “It is hoped that medical staff can share decision-making and discuss advance care planning with family members” (4.42 ± 0.78) and “I hope the older adult die peacefully and with dignity, surrounded by family members” (4.42 ± 0.66).

**Table 4:** Top five highest scoring items for family caregiver home hospice needs.

**Item**	**Dimension**	**Score ($\bar{X}\pm$SD)**
It is hoped that medical staff can share decision-making and discuss advance care planning with family members	End-of-life knowledge needs	4.42 ± 0.78
I hope the older adult die peacefully and with dignity, surrounded by family members	Spiritual care needs	4.42 ± 0.66
Hope to help me adjust the fear and sadness caused by the old man's death	Emotional regulation needs	4.40 ± 0.76
Hope to provide me with simple treatment guidance and emergency assistance at home when my condition changes or accidents occur (such as falling down from the bed or choking)	Life care needs	4.40 ± 0.73
It is hoped that institutions and medical staff will provide on-site formal care assistance (such as drug administration, disease monitoring services, and guidance for practical operations).	Social support needs	4.25 ± 0.65

### 3.3 Univariate analyses of family caregivers' home hospice needs

After testing the data for normality and homogeneity of variance, we screened for single factors that independently influenced the total FCNQ score and each dimension. The total score of needs showed that the duration of illness, number of comorbidities, self-care ability, age and education level of the dying older adult were statistically significant. Significant differences were also found in the age, relationship with the older adult, length of care, and caregiving experience of family caregivers. More detailed information is shown in Tables 5, 6.

**Table 5:** A univariate analysis of the characteristics of dying older adult with home hospice needs.

**Variable**	**SCN**	**LCN**	**ERN**	**SSN**	**EKN**	**SCN**	**TS**
**Older adults type of disease**							
Non-cancer chronic diseases	15.78 ± 3.53	23.95 ± 4.57	24.99 ± 5.07	16.95 ± 3.06	23.92 ± 2.73	14.63 ± 2.24	120.22 ± 16.15
Cancer	15.39 ± 3.25	24.74 ± 4.06	25.88 ± 4.79	18.18 ± 2.96	24.15 ± 2.69	14.61 ± 2.17	122.95 ± 14.24
*t/F*	0.873	−1.369	−1.346	**−3.058**	−0.638	0.045	−1.343
*p*	0.384	0.173	0.18	**0.003**	0.524	0.964	0.181
**Older adults duration of illness (year)**							
< 1	14.58 ± 3.39	23.88 ± 4.48	24.34 ± 5.21	16.62 ± 3.13	23.55 ± 2.84	14.21 ± 2.19	117.16 ± 15.97
1–3	15.67 ± 2.90	24.99 ± 4.21	26.02 ± 4.48	18.27 ± 2.82	24.30 ± 2.20	15.06 ± 2.06	124.32 ± 13.10
>3	16.54 ± 3.67	24.09 ± 4.27	25.91 ± 5.03	17.78 ± 3.05	24.23 ± 3.05	14.54 ± 2.31	123.09 ± 15.96
*t*/*F*	**6.258**	1.462	2.737	**6.093**	1.787	**3.050**	**4.891**
*p*	**0.002**	0.234	0.067	**0.003**	0.170	**0.049**	**0.010**
**Older adults number of comorbidities (kind)**							
1	15.23 ± 3.29	24.15 ± 4.46	24.91 ± 4.83	17.31 ± 3.10	23.83 ± 2.50	14.43 ± 2.13	119.88 ± 14.38
2	15.58 ± 3.13	24.14 ± 3.82	25.46 ± 5.27	17.54 ± 3.11	23.77 ± 2.82	14.59 ± 2.23	121.08 ± 15.21
≥3	16.71 ± 4.04	25.46 ± 4.83	27.14 ± 4.25	18.54 ± 2.73	25.26 ± 2.84	15.29 ± 2.30	128.40 ± 16.56
*t*/*F*	2.590	1.368	2.781	2.193	**4.370**	2.035	**4.393**
*p*	0.077	0.257	0.064	0.114	**0.014**	0.133	**0.013**
**Older adults self-care ability**							
Completely capable of	15.16 ± 2.90	24.59 ± 3.95	25.83 ± 4.92	18.22 ± 2.58	24.41 ± 2.34	14.66 ± 2.00	122.86 ± 12.98
Most able to	15.39 ± 3.28	23.58 ± 4.50	24.59 ± 5.08	17.23 ± 3.28	23.74 ± 2.59	14.43 ± 2.21	118.95 ± 15.32
A part of	15.60 ± 3.60	24.56 ± 4.39	25.38 ± 4.41	17.10 ± 3.10	23.36 ± 2.91	14.40 ± 2.00	120.40 ± 14.25
Completely unable	17.04 ± 4.05	25.89 ± 4.10	27.43 ± 5.01	18.21 ± 3.07	25.39 ± 2.91	15.54 ± 2.73	129.50 ± 18.54
*t*/*F*	2.152	2.252	2.556	2.081	**4.304**	2.032	**3.765**
*p*	0.095	0.083	0.056	0.104	**0.006**	0.243	**0.044**
**Older adults age (year)**							
60–69	15.77 ± 3.48	24.90 ± 4.30	26.07 ± 4.88	18.21 ± 2.97	24.17 ± 2.75	14.69 ± 2.31	123.82 ± 15.42
70–79	15.13 ± 3.27	23.78 ± 4.09	24.44 ± 5.17	17.03 ± 2.98	23.77 ± 2.55	14.42 ± 2.02	118.57 ± 13.53
≥80	16.07 ± 3.39	24.27 ± 4.77	26.05 ± 4.34	17.30 ± 3.23	24.27 ± 2.91	14.86 ± 2.32	122.82 ± 17.23
*t*/*F*	1.366	1.535	2.914	**3.638**	0.706	0.678	**2.887**
*p*	0.257	0.218	0.056	**0.028**	0.495	0.509	**0.047**
**Older adults education level**							
Primary school and below	15.37 ± 2.99	23.61 ± 4.11	24.87 ± 5.01	17.02 ± 3.07	23.78 ± 2.63	14.37 ± 2.06	119.02 ± 13.93
Middle school education	15.25 ± 3.73	24.33 ± 4.35	25.52 ± 5.03	17.78 ± 2.95	24.15 ± 2.66	14.58 ± 2.34	121.61 ± 16.40
High school education or technical secondary school	17.00 ± 4.47	26.54 ± 4.12	26.31 ± 4.77	18.69 ± 2.87	24.31 ± 3.88	15.31 ± 2.25	128.15 ± 14.75
Junior college and above	17.35 ± 3.45	28.29 ± 3.48	28.71 ± 2.42	20.12 ± 1.97	25.29 ± 2.14	16.12 ± 2.09	135.88 ± 11.38
*t*/*F*	2.74	**7.71**	**3.273**	**6.372**	1.705	**3.737**	**7.614**
p	0.104	**0.000**	**0.000**	**0.000**	0.167	**0.012**	**0.000**

SCN, symptom control needs; LCN, life care needs; ERN, emotional regulation needs; SSN, social support needs; EKN, end-of-life knowledge needs; SCN, spiritual care needs; TS, total score.

The bold indicated the *p* < 0.05.

**Table 6:** A univariate analysis of family caregiver characteristics for home hospice needs.

**Variable**	**SCN**	**LCN**	**ERN**	**SSN**	**EKN**	**SCN**	**TS**
**Gender**							
Male	14.86 ± 3.14	24.11 ± 4.46	25.27 ± 4.85	17.61 ± 3.22	23.98 ± 2.40	14.53 ± 2.11	120.35 ± 15.19
Female	16.02 ± 3.47	24.50 ± 4.25	25.55 ± 5.01	17.56 ± 2.98	24.07 ± 2.88	14.68 ± 2.26	122.37 ± 15.27
*t*/*F*	**−2.52**	−0.655	−0.406	0.12	−0.256	−0.484	−0.963
*p*	**0.012**	0.513	0.685	0.905	0.798	0.629	0.336
**Age (year)**							
18–40	16.3 ± 3.53	24.8 ± 4.58	26.59 ± 4.57	18.05 ± 3.25	24.81 ± 2.39	15.31 ± 2.31	125.88 ± 16.53
41–59	15.1 ± 3.25	24.2 ± 4.40	25.25 ± 4.94	17.64 ± 3.08	23.89 ± 2.71	14.49 ± 1.98	120.59 ± 14.14
≥60	15.6 ± 3.41	24.1 ± 4.03	24.75 ± 5.14	17.10 ± 2.86	23.59 ± 2.85	14.22 ± 2.29	119.45 ± 15.11
*t*/*F*	2.251	0.445	2.331	1.554	**3.523**	**4.293**	**3.254**
*p*	0.108	0.641	0.1	0.214	**0.031**	**0.015**	**0.04**
**Marital status**							
Unmarried	16.10 ± 4.61	25.20 ± 4.66	26.80 ± 5.87	18.10 ± 4.10	25.00 ± 3.16	15.40 ± 2.07	126.60 ± 22.09
Married/cohabitation	15.53 ± 3.34	24.29 ± 4.33	25.47 ± 4.80	17.56 ± 3.02	24.02 ± 2.65	14.59 ± 2.20	121.46 ± 14.91
Divorce	18.00 ± 1.41	26.50 ± 3.54	15.50 ± 6.36	17.50 ± 3.54	21.00 ± 5.66	13.50 ± 3.54	112.00 ± 7.07
*t*/*F*	0.645	0.46	**4.58**	0.15	1.915	0.902	0.944
*p*	0.526	0.632	**0.011**	0.861	0.15	0.407	0.351
**Education level**							
Junior high school and below	15.38 ± 3.08	23.95 ± 4.17	25.49 ± 4.58	17.20 ± 3.04	23.80 ± 2.58	14.37 ± 2.13	120.18 ± 13.93
High school or junior college	15.29 ± 3.42	24.80 ± 4.39	25.04 ± 5.28	17.44 ± 2.96	23.96 ± 2.98	14.93 ± 2.07	121.47 ± 16.19
College or above	16.46 ± 4.09	25.07 ± 4.65	25.70 ± 5.64	18.83 ± 2.97	24.78 ± 2.72	15.04 ± 2.45	125.87 ± 17.32
*t*/*F*	1.96	1.454	0.211	**5.072**	2.285	2.203	2.416
*p*	0.243	0.236	0.81	**0.007**	0.104	0.113	0.092
**Health status**							
Very good	15.42 ± 3.55	24.18 ± 4.32	24.28 ± 5.07	17.16 ± 3.18	24.27 ± 2.43	14.58 ± 2.30	119.89 ± 16.40
Normal	15.65 ± 3.25	25.27 ± 4.40	26.37 ± 4.12	18.06 ± 3.24	24.18 ± 2.99	14.89 ± 2.04	124.41 ± 14.40
Not good enough	15.77 ± 3.40	23.71 ± 4.15	25.90 ± 4.94	17.81 ± 2.62	23.81 ± 2.53	14.58 ± 1.93	121.58 ± 14.24
Poor	15.63 ± 3.38	23.13 ± 4.16	26.44 ± 6.48	17.13 ± 2.73	22.75 ± 3.19	13.75 ± 2.93	118.81 ± 14.41
*t*/*F*	0.129	1.914	**2.894**	1.352	1.619	1.2	1.364
*p*	0.943	0.128	**0.042**	0.258	0.186	0.311	0.255
**Caregiving burden**							
No burden of care	15.58 ± 3.90	23.36 ± 4.37	23.15 ± 6.11	17.27 ± 3.62	24.12 ± 2.95	14.82 ± 2.39	118.30 ± 18.02
A little burden of care	15.76 ± 3.29	24.85 ± 4.54	25.03 ± 4.40	17.83 ± 2.98	24.49 ± 2.49	14.94 ± 1.95	122.89 ± 15.21
Heavy burden of care	15.34 ± 3.34	24.07 ± 3.95	26.91 ± 4.69	17.38 ± 2.95	23.40 ± 2.79	14.12 ± 2.36	121.23 ± 13.99
*t*/*F*	0.357	1.776	**8.064**	0.692	**3.873**	**3.433**	1.189
*P*	0.7	0.172	**0.002**	0.502	**0.022**	**0.034**	0.306
**Religious belief**							
No	15.47 ± 3.22	24.23 ± 4.26	25.43 ± 4.94	17.49 ± 3.02	23.97 ± 2.68	14.53 ± 2.15	121.12 ± 14.63
Yes	16.94 ± 4.93	25.82 ± 4.94	25.59 ± 5.03	18.71 ± 3.46	24.82 ± 2.94	15.71 ± 2.57	127.59 ± 20.90
*t*/*F*	−1.21	−1.467	−0.125	−1.58	−1.251	**−2.133**	−1.252
*P*	0.243	0.144	0.902	0.115	0.212	**0.034**	0.227
**Relationship with the dying older adult**							
Spouse	14.66 ± 3.09	23.84 ± 3.77	24.99 ± 5.36	17.12 ± 2.92	23.52 ± 2.70	14.04 ± 2.23	118.17 ± 13.89
Children	16.10 ± 3.55	24.80 ± 4.68	26.04 ± 4.57	17.98 ± 3.11	24.52 ± 2.64	14.98 ± 2.11	124.43 ± 15.90
Grandchildren	14.57 ± 2.88	23.43 ± 3.60	23.14 ± 5.15	17.14 ± 3.39	23.86 ± 1.57	15.43 ± 1.99	117.57 ± 14.67
Sibling	15.89 ± 2.32	23.44 ± 3.71	23.22 ± 5.54	16.44 ± 3.54	22.11 ± 3.52	13.78 ± 2.33	114.89 ± 9.98
Other	17.40 ± 3.51	23.60 ± 4.98	24.60 ± 4.72	17.20 ± 1.79	23.60 ± 1.34	14.80 ± 2.28	121.20 ± 15.82
*t*/*F*	**2.779**	0.819	1.51	1.36	**2.974**	**2.875**	**2.702**
*P*	**0.028**	0.514	0.2	0.249	**0.02**	**0.024**	**0.031**
**Length of caregiving (month)**							
1–6	14.87 ± 2.85	24.21 ± 4.22	25.60 ± 4.20	17.47 ± 3.05	23.93 ± 2.54	14.54 ± 2.04	120.61 ± 13.76
6–12	15.92 ± 3.49	23.29 ± 3.91	24.19 ± 5.39	17.17 ± 2.96	23.81 ± 2.80	14.52 ± 2.11	118.90 ± 14.15
>12	16.62 ± 3.94	25.53 ± 4.63	26.23 ± 5.63	18.13 ± 3.15	24.43 ± 2.92	14.87 ± 2.55	125.82 ± 17.93
*t*/*F*	**5.79**	**3.977**	2.527	1.511	0.92	0.512	**3.423**
*P*	**0.006**	**0.02**	0.134	0.223	0.4	0.6	**0.034**
**Household incomes per capita(yuan)**							
< 3,000	15.32 ± 3.14	24.02 ± 3.98	26.22 ± 4.85	17.00 ± 3.09	23.71 ± 2.74	14.35 ± 2.23	120.63 ± 15.07
3,000–4,999	15.71 ± 3.43	24.55 ± 4.50	25.52 ± 4.47	18.10 ± 2.91	24.53 ± 2.55	14.83 ± 2.16	123.24 ± 15.25
≥5,000	15.88 ± 3.86	24.65 ± 4.73	23.58 ± 5.62	17.79 ± 3.18	23.74 ± 2.85	14.79 ± 2.20	120.44 ± 15.64
*t*/*F*	0.515	0.468	**4.369**	**3.112**	2.394	1.222	0.819
*P*	0.598	0.627	**0.014**	**0.046**	0.094	0.297	0.442
**Caregiving experience**							
No	15.24 ± 3.31	23.86 ± 4.25	25.17 ± 4.94	17.24 ± 3.00	23.89 ± 2.56	14.39 ± 2.12	119.80 ± 14.51
Yes	16.59 ± 3.46	25.80 ± 4.27	26.25 ± 4.87	18.59 ± 3.06	24.46 ± 3.08	15.32 ± 2.31	127.02 ± 16.19
*t*/*F*	**−2.604**	**−2.957**	−1.417	**−2.891**	−1.251	**−2.795**	**−3.129**
*P*	**0.01**	**0.003**	0.158	**0.004**	0.214	**0.006**	**0.002**
**Burden of payment for treatment**							
No burden	15.13 ± 3.48	24.27 ± 3.96	21.40 ± 5.99	18.47 ± 2.90	23.60 ± 2.53	14.53 ± 2.26	117.40 ± 14.55
A little burden	16.48 ± 3.92	25.30 ± 4.75	25.04 ± 4.93	18.43 ± 3.08	24.88 ± 2.78	15.48 ± 2.08	125.61 ± 17.22
Moderate burden	15.37 ± 3.23	23.98 ± 4.56	24.82 ± 4.50	17.24 ± 3.19	23.94 ± 2.53	14.69 ± 2.05	120.04 ± 14.75
Heavy burden	15.26 ± 3.10	24.02 ± 4.00	26.54 ± 4.65	17.15 ± 2.94	23.69 ± 2.71	14.13 ± 2.20	120.80 ± 14.18
*t*/*F*	1.801	1.235	**5.802**	**2.780**	2.559	**4.818**	1.963
*p*	0.148	0.298	**0.001**	**0.042**	0.056	**0.003**	0.120

SCN, symptom control needs; LCN, life care needs; ERN, emotional regulation needs; SSN, social support needs; EKN, end-of-life knowledge needs; SCN, spiritual care needs; TS, total score.

The bold indicated the *p* < 0.05.

### 3.4 Multiple linear regression analysis of family caregivers' need for home hospice care

Relationship with the dying older adult (spouse as the benchmark) and Caregiver's marital status (unmarried as the benchmark) of the independent variables were set as dummy variables. Other independent variable assignments are shown in Table 7.

**Table 7:** Regression analysis of factors influencing total need and need by dimension.

**Dimension**	**Variable^a^**	**Unstandardized coefficients**	**Standardized coefficients**	**Standardized coefficients**	***R* ^2^**	**Cumulative *R*^2^**	***t***	***p***
		β	**SE**	β				
SCN	Constant	8.385	1.156		0.188	0.158	7.251	0.000
	Relationship with the dying older adult (children)	1.723	0.461	0.253			3.736	0.000
	Caregiver's gender (female)	1.303	0.434	0.187			3.006	0.003
	Length of caregiving (>12 month)	0.780	0.261	0.195			2.988	0.003
	Older adults duration of illness (year)	0.771	0.267	0.181			2.886	0.004
LCN	Constant	19.878	0.999		0.117	0.105	19.889	0.000
	Caregiving experience (yes)	1.620	0.652	0.162			2.483	0.014
	Older adults education level	1.341	0.309	0.278			4.335	0.000
ERN	Constant	21.679	2.313		0.181	0.154	9.373	0.000
	Caregiver's marital status (divorce)	−12.1	3.642	−0.231			−3.322	0.001
	Caregiving burden (heavy burden of care)	1.573	0.560	0.218			2.807	0.005
	Older adults education level	1.102	0.343	0.200			3.210	0.002
SSN	Constant	12.403	1.620		0.209	0.180	7.673	0.000
	Caregiver's education level	0.543	0.255	0.143			2.128	0.035
	Caregiving experience (yes)	1.082	0.433	0.153			2.502	0.013
	Older adults type of disease	1.147	0.471	0.188			2.436	0.016
	Older adults duration of illness (year)	0.879	0.251	0.229			3.501	0.001
	Older adults education level	0.628	0.223	0.184			2.815	0.005
EKN	Constant	24.453	1.144		0.098	0.064	21.373	0.000
	Caregiving burden	0.527	0.265	0.133			1.988	0.048
	Older adults number of comorbidities (kind)	0.622	0.249	0.17			2.495	0.013
SCN	Constant	13.446	1.183		0.165	0.122	11.367	0.000
	Caregiving experience (yes)	0.863	0.325	0.170			2.659	0.008
	Older adults education level	0.446	0.158	0.182			2.821	0.005
TS	Constant	100.368	6.166		0.227	0.183	16.277	0.000
	Caregiving experience (yes)	5.794	2.235	0.165			2.593	0.010
	Older adults duration of illness (year)	2.664	1.304	0.14			2.043	0.042
	Older adults education level	4.511	1.07	0.266			4.217	0.000

SCN, symptom control needs; LCN, life care needs; ERN, emotional regulation needs; SSN, social support needs; EKN, end-of-life knowledge needs; SCN, spiritual care needs; TS, total score.

^a^Dummy variables: relationship with the dying older adult (spouse) = 0, Caregiver's gender (male) = 0, length of caregiving (1–6 month) = 0, older adults duration of illness (< 1 year) = 0, Caregiving experience (no) = 0, older adults education level (primary school and below) = 0, caregiver's marital status (unmarried) = 0, caregiving burden (no burden of care) = 0, older adults type of disease (non-cancer chronic diseases) = 0, older adults number of comorbidities (1 kind) = 0.

Seven multivariate linear regression analyses were performed to identify significant correlates of total home hospice needs and six dimensions of needs. The diagnostic test independent variables had all variance inflation factor (VIF) values < 10, indicating no multicollinearity issue existed before the analysis was conducted. Furthermore, residual analysis provided support for the equation models' linearity, normality, and homogeneity of variance. Caregivers with caregiving experience (β = 5.794, *P* = 0.010) as well as dying older adult reporting longer duration of illness (β = 2.664, *P* = 0.042), and higher levels of education (β = 4.511, *P* = 0.000) had more total home hospice need for family caregivers, with 22.7% of the variance explained. Caregiver being female (β = 1.303, *P* = 0.003), longer duration of caregiving (β = 0.780, *P* = 0.003), relationship with the older adult was children (β = 1.723, *P* = 0.000), and longer duration of illness for the dying older adult (β = 0.771, *P* = 0.004) were all associated with caregiver symptom control needs, with 18.8% of the variance explained. The influence factors of the other dimensions are shown in Table 7.

## 4 Discussion

### 4.1 Family caregivers' home hospice care needs

The results of this study indicate that there is a high level of hospice needs for the family caregivers of the older adult at the end of life with chronic diseases in the home setting. First, in the current study, we found that family caregivers of terminally ill older adult people with end-stage chronic diseases have different levels of needs in all six dimensions. Family caregivers had the highest dimension level of end-of-life knowledge needs, where the highest scoring items corroborate that in China, most of the treatment decisions and advance care planning are made by family members in consultation with medical professionals, consistent with the findings of the study by Gu et al. (22). China has a long history of family-centered collectivist culture, and the family serves the functions of emotional connection, communication, and dealing with life's challenges. Family members are crucial in therapeutic communication considering the physical state and psychological tolerance of the older adult.

Secondly, spiritual care needs are more prominent. Among them, accompanying the older adult peacefully and passing away is considered to be the most important need, which is consistent with the survey of Chinese scholars (23). This means that many family members avoid talking to the patients about their illnesses and cannot truthfully inform the patients of the fact that they have reached the terminal stage. As a result, some patients have unrealistic expectations for how their illnesses will be treated, making it difficult for them to pass away peacefully and dignifiedly.

Thirdly, emotional regulation was also identified as a high need in this study, of which the item of regulating the emotion of fear and sadness was the most important, indicating that the fear and helplessness of the family members increase significantly during the dying period and when the patient's condition deteriorates (16). During the implementation of home hospice care, healthcare professionals should pay attention to the psychological state of family caregivers, provide them with timely psychological support, guide them to cope with negative emotions and establish the correct confidence in caregiving. Symptom control needs have the lowest scores, which belong to the lowest physiological needs in Maslow's hierarchy of needs theory and should be met first (24). This implies that medical professionals in China's existing healthcare system concentrate on managing symptoms. For example, Jing'e team's core service for home hospice is analgesic treatment with guidance on symptom control (25), which shows that the need for symptom control has been largely met.

### 4.2 Influencing factors of home hospice care needs

Next, we identified several factors related to the home hospice care needs of family caregivers of older adult people with terminal chronic illnesses. We discovered that family caregivers with caregiving experience had high home hospice care needs, particularly for life care needs, social support needs, and end-of-life knowledge needs, which was consistent with the study by Wei et al. (26). Experienced family caregivers witnessed patients suffering from symptoms such as pain, nausea, vomiting, anxiety, and depression, prompting a stronger need for daily life care. They hoped that the care would allow the terminally ill older adult to gain somatic comfort. In addition, the older adult dying in this study had comorbidities and 74.1% had self-care deficits. The complexity and variety of older adult conditions made it urgent for even seasoned caretakers to seek professional help. According to Jansma, caregivers who have caregiving experience are mentally ready for the patient's death and want to learn more about end-of-life knowledge in advance so that the deceased can rest in peace and the living can be eased (19). Therefore, the focus of healthcare professionals' knowledge and skills in teaching and instructing also includes caregivers who have experience in caregiving, so that caregivers are able to cope with various emergencies that may arise in family caregiving in an organized manner and meet the needs. However, how healthcare professionals can use different teaching methods and curricula to target caregivers at different levels of care is a topic that needs to be further explored.

This study identified gender as an influential factor in caregivers' symptom control needs. This validates Franchini et al.'s findings (27). With 62.1% of the caregivers in this survey being female, women dominated the role of caregiver due to the Chinese idea that men work hard for a career and women manage the household. Women are more sensitive and thoughtful, more capable of fulfilling the caregiver role and independently accomplishing illness and life care, which leads to their higher needs. In contrast, Lili et al. reported in a stress load survey of primary caregivers of patients with advanced primary liver cancer that women had high load scores (28). Therefore, male family members should be encouraged to actively participate in the care of the older adult by providing guidance and assistance to improve their caregiving skills and quality of care.

The study's caregivers were primarily young and middle-aged, ranging in age from 41 to 59 (43.3%), with most of them being the older adult terminally ill patients' children (56.5%). This group is mostly the economic and spiritual pillars of the family, with jobs and children, and the need to bear the pressure from family, work and study, as well as the task of long-term caregiving with a focus on symptom control. Therefore, the need for caregivers whose relationship with the dying older adult is that of a child and who take care of them for a longer time was higher. In addition, prolonged heavy caregiving is associated with a range of physical and psychological problems, leading to a decline in the quality of care (29). It is fully consistent with the finding of this study that the higher the caregiving burden, the higher the emotional regulation needs and spiritual care needs of caregivers. Consequently, healthcare professionals should pay special attention to the emotional and spiritual states of caregivers in this age group in the process of providing guidance to family caregivers, as well as understand their needs for palliative care in a timely manner and provide professional explanations.

According to this study, family caregivers' needs for home hospice care increased with the length of the older adult patient's illness, especially for symptom control needs, social support needs and spiritual care needs. Oechsle et al. reported that the psychological state of the primary caregiver of a cancer patient worsened as the patient's condition deteriorated and functional status declined (30). Because of the older chronic disease group's longer illness duration and the disease's gradual worsening of symptoms, their carers start to consider matters pertaining to death, which exacerbates emotional distress like anxiety and sadness. The retrospective study by Cengiz et al. also demonstrated that as caregiving grew longer and the patient's condition worsened, the caregiver's needs became more complex and comprehensive, necessitating more medical services to relieve the patient's physical and emotional suffering (31). Patients with malignant tumors confront significant physical, psychological, and spiritual challenges, according to research by Won et al. (32). The vast majority of terminal cancer patients are in critical condition, extremely weak and fatigued, with severely reduced mobility and self-care ability. They require caregiver support even for basic tasks like turning over in bed and defecating (33). However, this survey found that the percentage of those with no caregiving experience was as high as 75.0%, so caregivers are in great need of information and emotional support from family members, especially from other healthcare providers. In line with the findings of this study, family caregivers of terminally ill older adult with cancer had high social support needs.

In this study, caregiver needs were also influenced by education level. Family caregivers' total needs for home hospice care—which include life care needs, emotional regulation needs, social support needs, and spiritual care needs—increased with the terminally ill older adult' educational level. The higher literacy level of the terminally ill means better cognition and higher acceptance of hospice care (34). They have a greater desire to use home hospice care to enhance their quality of life when they are dying. Older adult people and their families live in the same household, and the long time together makes their life concepts and values converge (35). The old person's wishes to die at home will be honored by the caregivers. Additionally, this study also found that the higher education level of caregivers was associated with higher social support needs. Longacre maintained that the more educated the caregivers were, the more they wanted to utilize resources to access health care services and information (36). The reason for this is that highly educated caregivers are more demanding in their work and lives and are strict about improving quality care for their patients. They need to know or learn more information and knowledge, so they need more outside guidance and support. For family caregivers with higher education levels, they can provide more in-depth and professional knowledge about home hospice care, and recommend them to go through professional books, websites and public numbers to increase their rational understanding and participate in hospice care together.

## 5 Limitations

This study has the following limitations. First, this study was conducted in Jinzhou City, Liaoning Province, which is useful for studies under the same economic and cultural conditions, but whether the conclusions are representative of family caregivers of the chronically dying older adult in other regions needs to be further explored, and future studies could increase the sample size and collect data in different regions to make the findings more generalizable. Second, this study was a cross-sectional survey that investigated the need for and factors influencing home hospice care for this group at a certain point in time. In the future, a longitudinal study could be conducted to investigate the trends in caregivers' needs for home hospice care and the factors that influence them as the conditions of the terminally ill older adult change, so that better clinical guidance can be provided.

## Data availability statement

The original contributions presented in the study are included in the article/supplementary material, further inquiries can be directed to the corresponding author.

## Ethics statement

The studies involving humans were approved by Jinzhou Medical University College of Nursing Ethics Committee. The studies were conducted in accordance with the local legislation and institutional requirements. The participants provided their written informed consent to participate in this study.

## Author contributions

LW: Writing – original draft. YL: Writing – original draft. RZ: Writing – review & editing. JL: Writing – original draft. XG: Writing – original draft. HL: Writing – review & editing.
